# Correlation between toxic organic acid fluctuations and neurodevelopment in patients with methylmalonic acidemia

**DOI:** 10.1186/s13023-025-03687-3

**Published:** 2025-04-15

**Authors:** I.-Chih Ling, Dau-Ming Niu, Chia-Feng Yang, Cheng-Yu Lee, Sheng-Bin Liang, Yann-Jang Chen

**Affiliations:** 1 Department of Pediatrics, Taipei Veterans General Hospital, Taipei, Taiwan; 2 Department of Pediatrics, Wan Fang Hospital, Taipei Medical University, Taipei, Taiwan; 3 Institute of Clinical Medicine, School of Medicine, National Yang-Ming Chiao Tung University, Taipei, Taiwan; 4 Department of Pediatrics, Cardinal Tien Hospital, New Taipei City, Taiwan; 5 Department of Life Sciences and Institute of Genome Sciences, National Yang-Ming Chiao Tung University, 155 Linon Street, Sec 2, Beitou, Taipei, 11221 Taiwan; 6 Department of Education and Research, Taipei City Hospital, Taipei, Taiwan

**Keywords:** Methylmalonic acidemia, Methylmalonic acid, Methylcitric acid, Mutase, Cobalamin, Neurodevelopment

## Abstract

**Background:**

Methylmalonic acidemia (MMA) is a rare autosomal recessive disorder, that causes multisystem damage by accumulating toxic metabolites. These metabolites, particularly affecting nerve cells, contribute to suboptimal neurodevelopment in MMA patients. While fluctuations in these toxic metabolites are common in MMA patients, their precise impact on neurodevelopment remains unclear.

**Results:**

This study enrolled 20 MMA patients, comprising 14 vitamin B12 non-responsive (B12-NR) type and 6 vitamin B12 responsive (B12-R) type. Diverse parameters were assessed, including methylmalonic acid (MA), methylcitric acid (MCA), propionylcarnitine (C3), acetylcarnitine (C2), ammonia, glycine, and lactate. Cognitive function was evaluated using the Bayley-III and Wechsler intelligence scale, and brain imaging was conducted through magnetic resonance spectroscopy (MRS). The frequency and extent of fluctuations in toxic organic acids were computed based on blood test results. B12-NR type patients exhibited elevated levels of MA, MCA, C3, C3/C2 ratio and lactate, with more frequent and significant MA, MCA and C3 fluctuation than B12-R type patients. Brain imaging revealed central nervous system demyelination in B12-NR type patients, while B12-R type patients displayed normal MRS results. B12-R type patients exhibited significantly better neurocognitive outcomes, with higher scores in all domains.

**Conclusion:**

Patients with B12-NR type MMA exhibit worse neurodevelopmental outcomes and more pronounced biochemical imbalances compared to those with B12-R type. Significant correlations were observed between higher fluctuation frequencies of toxic metabolites and lower developmental and IQ scores. These findings emphasize the importance of targeted strategies to manage organic acid fluctuations for improving neurodevelopmental outcomes in MMA.

**Supplementary Information:**

The online version contains supplementary material available at 10.1186/s13023-025-03687-3.

## Background

Methylmalonic acidemia (MMA) is a rare and clinically diverse autosomal recessive disorder characterized by a deficiency in methylmalonyl-CoA mutase, leading to the accumulation of harmful metabolites [1, 2]. The primary genetic defects behind MMA are mutations in the MMUT gene, responsible for encoding methylmalonyl-CoA mutase, or mutations in genes involved in cobalamin metabolism, crucial for adenosylcobalamin generation–a vital cofactor of mutase [3, 4].

Biochemical analysis plays a pivotal role in MMA diagnosis, with detectable elevations in methylmalonic acid (MA) and methylcitric acid (MCA) levels serving as key indicators [1, 2]. This accumulation disrupts regular cellular processes, contributing to the spectrum of clinical manifestations, varying from severe to fatal [2]. Elevated propionylcarnitine (C3) levels and the ratio of propionylcarnitine to acetylcarnitine (C2) are significant markers in newborn screening programs, facilitating early detection of MMA [5]. Increased ammonia levels in MMA result from interconnected mechanisms, with MMA-induced oxidative stress impeding urea cycle enzyme functionality, and hindering ammonia clearance [6]. The endpoint of MA metabolism is the generation of succinyl-CoA, an important intermediate of the tricarboxylic acid (TCA) cycle. However, disrupted MA metabolism can interfere with the TCA cycle, potentially resulting in elevated lactate levels [6–8].

According to treatment responses to vitamin B12, MMA can be divided into vitamin B12 responsive (B12-R) and vitamin B12 non-responsive (B12-NR) subtypes. B12-NR MMA involves protein restriction, carnitine supplementation for organic acid clearance, and medication for ammonia removal. Severe cases may warrant liver transplantation. In contrast, B12-R MMA primarily requires vitamin B12 supplementation, often with hydroxocobalamin or derivatives, and a less strict protein-restricted diet [9–11]. Despite having distinct genetic origins and treatments, B12-NR and B12-R MMA share commonalities in biochemical markers and symptoms. Key biomarkers such as MA, MCA, and C3 are specific indicators used to monitor and evaluate treatment responses in both types [2, 9]. Both variants of MMA impact multiple organ systems, including neurological, gastrointestinal, hematological, cardiovascular, and renal systems [2, 12–17]. Neurological manifestations, particularly affecting development and cognition, significantly contribute to the clinical burden of MMA, ranging from mild developmental delays to severe cognitive impairment and neurological deficits, highlighting the crucial role of toxic organic acid accumulation in B12-NR and B12-R MMA patients [7, 13].

This study aims to comprehensively review and compare neurodevelopmental outcomes, brain imaging data, and biochemical markers in our cohort of MMA patients in Taiwan. Emphasis is placed on exploring the variability in toxic organic acid levels and quantifying its correlation with neurocognitive outcomes. Specifically, we investigate whether greater fluctuations in organic acid levels correspond to poorer neurodevelopmental outcomes, a facet not explored in existing literature.

## Methods

### Study population

This study enrolled a total of 20 MMA patients, with 14 individuals exhibiting defects in methylmalonyl-CoA mutase, and the remaining 6 patients having defects in cobalamin metabolism. The 14 patients with MMUT defects showing no response to hydroxycobalamin were classified as the B12-NR subgroup. The remaining 6 patients had defects in cobalamin metabolism, including 2 siblings with cbl-B type and 4 with cbl-C type, and were classified as B12-R subgroup, showing more than a 50% decrease in MA levels after hydroxycobalamin treatment. Under the use of betaine, L-carnitine, and hydroxycobalamin, the four cbl C-type MMA patients showed stable homocysteine levels with a mean of 23.1 µmol/L. All had levels below 45 µmol/L, and no microvascular disease was noticed. All these 20 patients were diagnosed within 3 weeks after birth, due to the efficacy of our nationwide newborn screening policy. The follow-up period averaged 85 months, with a median of 84 months.

B12-NR patients are treated with protein restriction, L-carnitine supplementation, and sodium benzoate. In cases where patients encountered highly fluctuating serum ammonia levels and demonstrated failure to thrive due to malnutrition, liver transplantation (LT) was considered as one of the available treatment options. Additionally, B12-R patients received hydroxycobalamin as part of their treatment regimen.

### Biochemical markers

Patients with MMA were assessed for various biomarkers, including MA, MCA, C3, C3/C2, ammonia, glycine, and lactate during each visit. To gauge the fluctuation of these biomarkers, we initially examined concentration of each biomarker in serum or urine for each among the 20 MMA patients. The fluctuating event was defined as the absolute difference value between two blood test results taken six months apart, which represents the range of metabolite fluctuation between visits. We then calculate the standard deviation (SD) of the fluctuating events for each toxic metabolite, including MA, MCA, C3 and C3/C2. A “positive widely fluctuating event” is defined as a metabolite’s absolute difference value exceeding one SD, which is 155.5 μmol/mmol CRE, 6.1 μmol/L, 10.7 μM, and 0.45 for MA, MCA, C3, and C3/C2 respectively. The total number of fluctuation events for each biomarker was quantified and then normalized to the total number of blood tests conducted for each patient.

### Cognitive function tests

Various cognitive function assessments were administered based on the patient’s chronological age. The Bayley-III cognitive tests were utilized for individuals aged 0–36 months, the Wechsler Preschool Scale for those aged 36–72 months, and the Wechsler Intelligence Quotient (IQ) IV for those exceeding 72 months. Eight patients underwent the Bayley-III screening test, while six patients participated in the Wechsler Intelligence scale test. Developmental delay was defined as a Bayley III composite score below 85. In the Wechsler intelligence scale test, a score between 55 and 70 indicated mild cognitive developmental impairment, 40–55 as moderate impairment, 25–40 as severe impairment, and a score below 25 as profound impairment.

### Analysis of brain imaging

Magnetic resonance spectroscopy (MRS) was conducted to assess potential metabolic irregularities in the brain.

### Statistical analysis

Quantitative data were presented as means ± SD, and categorical variables were summarized accordingly. Based on the characteristics of our study, we have chosen specific statistical tests. To compare the two independent groups (B12-R and B12-NR), we utilized the unpaired *t *test because the data are continuous, normally distributed, and consist of independent samples. A *p*-value of less than 0.05 in the unpaired *t *test is considered statistically significant. This study involved several statistical comparisons to explore potential associations between biochemical markers and neurodevelopmental outcomes in patients with methylmalonic acidemia. Due to the rare nature of this disease and our small sample size, we chose not to apply adjustments for multiple comparisons. Pearson correlation analysis was employed to determine the potential correlation coefficient between developmental delay and fluctuations in toxic organic acids in MMA patients. Strong correlation is defined as |r| being between 0.7 and 1.

## Results

### Study population

This study included 20 participants, with a distribution of 9 boys and 11 girls, encompassing 14 B12-NR and 6 B12-R MMA cases. The average age of the patients was 6.8 ± 5.6 years. Within the 14 B12-NR individuals, eight had undergone LT at an average age of 1.5 ± 2.1 years. Additionally, among the 6 B12-R patients, there were 2 siblings with cbl-B type MMA and 4 with cbl-C type MMA (Table 1). However, Case 9 experienced recurrent hyperammonemia episodes, liver dysfunction, and jaundice. Subsequent pathological examination of his transplanted liver revealed the presence of Dubin-Johnson syndrome. Due to these co-morbidities after liver transplantation, he was excluded from the dataset for group comparisons in Tables 4, and 5.

**Table 1:** The clinical features of the twenty subjects in this study

Case no	Gender^a^	Age^b^ (years)	Subtype	Age at LT^c^ (years)	Zygosity^d^	Mutation type
1	F	8	B12-R	–	H	MMAB: c.523G > A,p.Gly175Ter
2	M	0.33	B12-R	–	H	MMAB: c.523G > A,p.Gly175Ter
3	M	10	B12-R	–	CH	MMACHC:c.609G > A,p.Trp203Ter MMACHC:c.658_660delAAG,p.Lys220del
4	M	7	B12-R	–	CH	MMACHC:c.228_231delTGAC,p.Asp77Glnfs*22 MMACHC: c.394C > T,p.Arg132Ter
5	F	3	B12-R	–	CH	MMACHC:c.609G > A,p.Trp203Ter MMACHC:c.626dupT,p.Thr210Aspfs*35
6	M	1	B12-R	–	CH	MMACHC:c.482G > A, p.Arg161Gln MMACHC:c.398_399del,p.Gln133Argfs*4
7	F	20	B12-NR	–	CH	MMUT:c.919 T > C,p.Phe307Leu MMUT:c.1280G > A,p.Gly427Asp
8	F	17	B12-NR	6.75	H	MMUT:c.1280G > A,p.Gly427Asp
9	M	14	B12-NR	0.67	H	MMUT:c.982C > T,p.Leu328Phe
10	F	11	B12-NR	0.67	CH	MMUT:c.454C > T,p.Arg152Ter MMUT:c.1677-1G > A
11	F	9	B12-NR	1	CH	MMUT:c.1159 A > C,p.Thr387Ile MMUT:c.1630_1631 GG > TA,p.Gly544Ter
12	M	9	B12-NR	0.92	CH	MMUT:c.729_730insTT,p.Asp244Leufs*39 MMUT:c.982C > T,p.Leu328Phe
13	F	8	B12-NR	1.17	H	MMUT:c.1280G > A,p.Gly427Asp
14	M	6	B12-NR	–	CH	MMUT:c.1105C > T,p.Arg369Cys MMUT:c.1280G > A,p.Gly427Asp
15	F	4	B12-NR	–	CH	MMUT:c.1280G > A,p.Gly427Asp MMUT:c.323G > A, p.Arg108His
16	M	2	B12-NR	0.5	CH	MMUT:c.1280G > A,p.Gly427Asp MMUT:c.1782_1786delTAAAG,p.Ser594Argfs*11
17	F	2	B12-NR	0.5	CH	MMUT:c.1280G > A,p.Gly427Asp MMUT:c.1782_1786delTAAAG,p.Ser594Argfs*11
18	F	2	B12-NR	–	CH	MMUT:c.1192A > C,p.Thr398Pro MMUT:c.454C > T,p.Arg152Ter
19	M	2	B12-NR	–	CH	MMUT:c.1655C > T,p.Ala552Val MMUT:c.1655C > T,p.Ala552Val
20	F	0.33	B12-NR	–	CH	MMUT: c.1106G > A,p.Arg369His MMUT:c.1677-1G > A

^a^F, female; M, male; ^b^Age at data collection; ^c^LT, liver transplantation; ^d^H, homozygous; CH, compound heterozygous

### Neurocognitive outcome in MMA patients

The Bayley III screening test was administered to two B12-R and six B12-NR patients younger than 3 years. Upon comparing the percentile rank (PR) values across cognitive, motor, language, and social-emotional scales, it was observed that B12-R patients displayed more favorable developmental outcomes than their B12-NR counterparts. The average PR values for the cognitive, motor, language and social-emotional scales in B12-NR patients were below 50. Except for two B12-NR patients (Case 12, 17) who demonstrated normal motor development, all other B12-NR patients experienced a global developmental delay. Furthermore, one B12-R type patient (Case 5) was classified as having developmental delay in cognitive functions, as evidenced by a Bayley-III composite score (CS) of 80 and a PR value of 9 for cognitive function (Table 2).

**Table 2:** Bayley-III screening test results for MMA patients

Case no	Subtype	Cognitive development and scale PR^#^	Motor development and scale PR	Language development and scale PR	Social emotional development and scale PR
4	B12-R	Normal (98)	Normal (88)	Normal (80)	Normal (99.6)
5	B12-R	Delay (9)	Delay (32)	Delay (34)	Normal (75)
11	B12-NR	Delay (5)	Delay (12)	Delay (13)	Delay (9)
12	B12-NR	Delay (14)	Normal (68)	Delay (0.1)	Delay (15)
13	B12-NR	Delay (1)	Delay (0.1)	Delay (3)	Delay (16)
14	B12-NR	Delay (0.1)	Delay (0.1)	Delay (0.1)	Delay (3)
16	B12-NR	Delay (5)	Delay (10)	Delay (1)	Delay (7)
17	B12-NR	Delay (5)	Delay (30)	Delay (5)	Delay (16)

^#^PR, percentile rank; number in parentheses indicates the PR value

Two B12-R and four B12-NR patients underwent the Wechsler Intelligence scale test. The test results indicated that B12-R patients achieved higher scores in terms of full-scale IQ (FSIQ), verbal IQ, and performance IQ. Specifically, the average FSIQ score was 85 (PR value 14.5) for B12-R patients and 63 (PR value 6.8) for B12-NR patients (Table 3). Among B12-NR patients, only one individual (case 9) was diagnosed with profound intellectual disability. At the age of 11, his FSIQ score was 22.

**Table 3:** Wechsler Intelligence scale test results for MMA patients

Case no	Subtype	Full scale IQ and PR^#^	Verbal IQ and PR	Performance IQ and PR	Intellectual disability
1	B12-R	84 (14)	89 (23)	80 (9)	–
3	B12-R	86 (15)	90 (23)	90 (15)	–
7	B12-NR	78 (10)	80 (13)	76 (9)	–
8	B12-NR	74 (5)	76 (5)	72 (4)	–
9	B12-NR	22 (1)	23 (1)	21 (1)	Profound
10	B12-NR	79 (11)	79 (12)	79 (10)	–

^#^PR, percentile rank; number in parentheses indicates the PR value

### Brain imaging findings in MMA patients

**Fig. 1:**
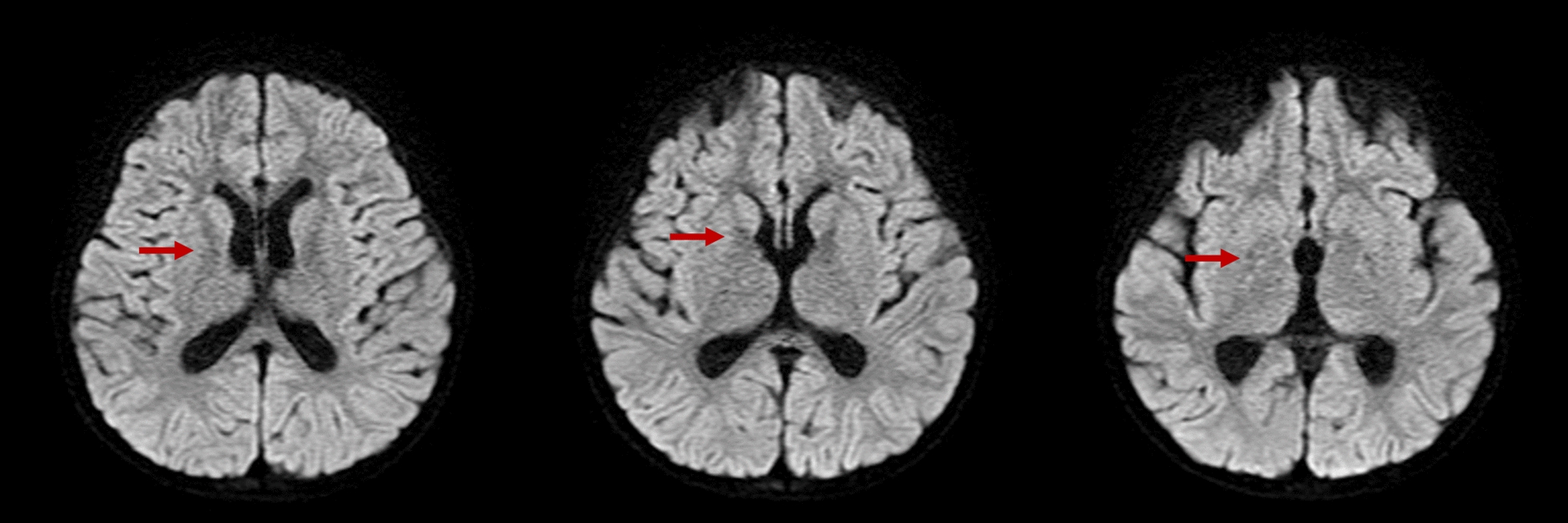
Brain MRI image of Case 9 at 7-years-old (DWI, axial view.). The arrows highlight the regions of encephalomalacia, which are prominently located in the bilateral lentiform nucleus, particularly affecting the globus pallidus

Fifteen patients, consisting of 11 B12-NR and 4 B12-R, underwent brain MRS studies at an average age of 4.8 ± 3.3 years. Elevated levels of choline complex with white matter demyelination were observed in 11 B12-NR patients, eight of whom had undergone liver transplantation. In contrast, all four B12-R patients exhibited normal brain MRS results. In our study population, 100% of B12-NR MMA patients exhibited brain MRS abnormalities, whereas 0% of B12-R MMA patients did. Despite liver transplantation (LT), brain demyelination was unavoidable, with 100% of B12-NR MMA patients who had undergone LT still showing brain demyelination. These findings may be limited by the small sample size. Details of the MRS findings in our study group are provided in Table S1. The brain MRS results for case 9, conducted at the age of 7, revealed not only white matter demyelination but also indications of prior injury, including patches of encephalomalacia and tissue loss in bilateral globus pallidi. Notably, case 9 was the sole individual with evident encephalomalacia on the brain image (Fig. 1). None of our patients displayed signs of optic nerve atrophy in their brain MRS examinations.

### Comparing in B12-R and B12-NR MMA Patients

The comparison of biochemical markers between patients with B12-R and B12-NR revealed notable elevations in MA, MCA, and C3 levels in the B12-NR group. However, the level of ammonia, lactate, C3/C2, and glycine showed no significant differences between these two groups (Table 4).

**Table 4:** Biochemical markers between B12-R and B12-NR patients

Patient	B12-R	B12-NR	*P* value
Serum ammonia (ug/dL)	53.4 ± 20.9	71 ± 31.6	0.17
Serum lactate (mg/dL)	40.4 ± 10.5	31.8 ± 18.4	0.17
Urine MA^a^ (μmol/mmol CRE)	69.5 ± 23.9	244.6 ± 66.4	0.002
Urine MCA^b^ (μmol/L)	2.82 ± 3.5	16.1 ± 10.4	0.007
Glycine (μmol/L)	238.8 ± 151.9	297.3 ± 110.9	0.39
C3^c^ (μM)	7.0 ± 3.9	31.8 ± 14.8	0.0003
C3/C2^d^	0.6 ± 0.42	0.77 ± 0.39	0.44

^a^MA, methylmalonic acid; ^b^MCA, methylcitric acid; ^c^C3, propionylcarnitine; ^d^C2, acetylcarnitine

In addition, a within-group comparison was conducted within the B12-NR group. Excluding Case 9, who experienced multiple complications after LT, the remaining seven B12-NR patients who underwent liver transplantation were compared with six B12-NR patients who did not. However, results showed no significant differences between the two groups (Table S2).

Notably, Case 5, the sole B12-R patient with delayed neurocognitive development in the Bayley III screening test, exhibited concentration levels of MA (76 μmol/mmol creatinine), MCA (4.48 μmol/L), and C3/C2 ratio (1.04) higher than those of another B12-R patient (Case 4) who demonstrated completely normal neurocognitive development. Case 4 had MA levels of 16.6 μmol/mmol creatinine, MCA levels of 0.45 μmol/L, and a C3/C2 ratio of 0.23 (Table S3). Furthermore, Case 5 displayed the highest levels of MA, MCA, and C3/C2 among all B12-R patients. Additionally, Case 9, the only B12-NR patient diagnosed with profound intellectual disability, showed elevated serum ammonia, averaging 125 μg/dL, in contrast to the average levels observed in other B12-NR patients (Table S3).

B12-NR patients demonstrated significantly higher frequencies of widely fluctuating MA, C3 and C3/C2 levels compared to B12-R patients. Moreover, B12-NR patients exhibited greater absolute differences in MA, MCA, and C3 between the two blood test results compared to B12-R patients (Table 5). In addition to the t-test, an ANOVA study was conducted to compare the two groups, and the results were consistent with the t-test findings. (Table S4) Within the B12-NR group, excluding Case 9 due to post-LT complications, no significant differences were found between the remaining seven patients who underwent liver transplantation and the six who did not. (Table S5).

**Table 5:** Variability in biomarker levels between B12-R and B12-NR patients

	B12-R	B12-NR	*p* value
Frequency of widely fluctuating MA #	0.14 ± 0.2	0.58 ± 0.29	0.01
Average MA fluctuation range (μmol/mmol CRE)	28.6 ± 33.6	147.9 ± 105.1	< 0.0001
Frequency of widely fluctuating MCA	0.07 ± 0.15	0.2 ± 0.18	0.15
Average MCA fluctuation range (μmol/L)	2.8 ± 3.41	5.24 ± 3.6	< 0.0001
Frequency widely fluctuating C3	0.07 ± 0.09	0.3 ± 0.21	0.01
Average C3 fluctuation range (μM)	3.2 ± 5.07	9.7 ± 6.12	< 0.0001
Frequency widely fluctuating C3/C2	0.04 ± 0.06	0.17 ± 0.13	0.01
Average C3/C2 fluctuation range	0.32 ± 0.7	0.29 ± 0.31	0.79

MA, methylmalonic acid; MCA, methylcitric acid; C2, acetylcarnitine; C3, propionylcarnitine

^#^ Frequency of wide fluctuation for each biomarker is normalized to total number of blood tests

In the case of Case 5, the only B12-R patient displaying a delay in neurocognitive development, there was a higher frequency of widely fluctuating MA (0.5 per blood test result), MCA (0.33 per blood test result), and C3 (0.17 per blood test result) compared to Case 4, who exhibited completely normal neurocognitive development. Case 4 demonstrated lower frequencies of widely fluctuating MA (0.06 per blood test result), MCA (0 per blood test result), and C3 (0 per blood test result). Notably, Case 5 displayed the highest frequency of widely fluctuating MA, MCA, and C3 among the entire group of B12-R patients (Table S3).

### Correlations between metabolite fluctuations and neurocognitive outcomes

**Fig. 2:**
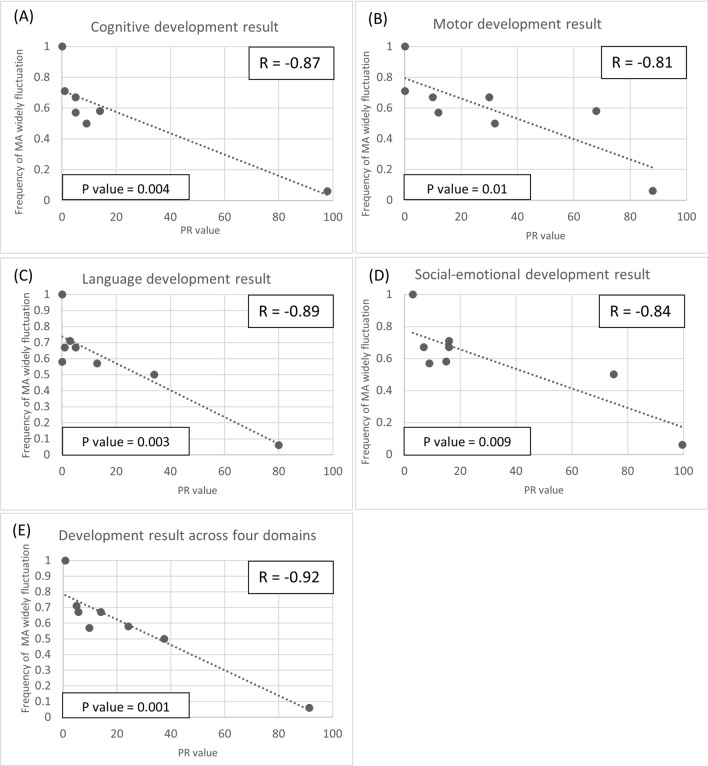
Scatter plot of PR values from the Bayley III screening test at different frequencies of MA fluctuation (> 1SD). Each dot represents an individual’s mean frequency of widely fluctuating MA per blood test and their corresponding developmental PR value. **A** Cognitive development result, **B** motor development result, **C** language development result, **D** Social-emotional development result, **E** average development result across all four domains. Significant correlations were identified between the widely fluctuating MA levels and all four domains of development, with each showing statistical significance at *p*-values below 0.05

**Fig. 3:**
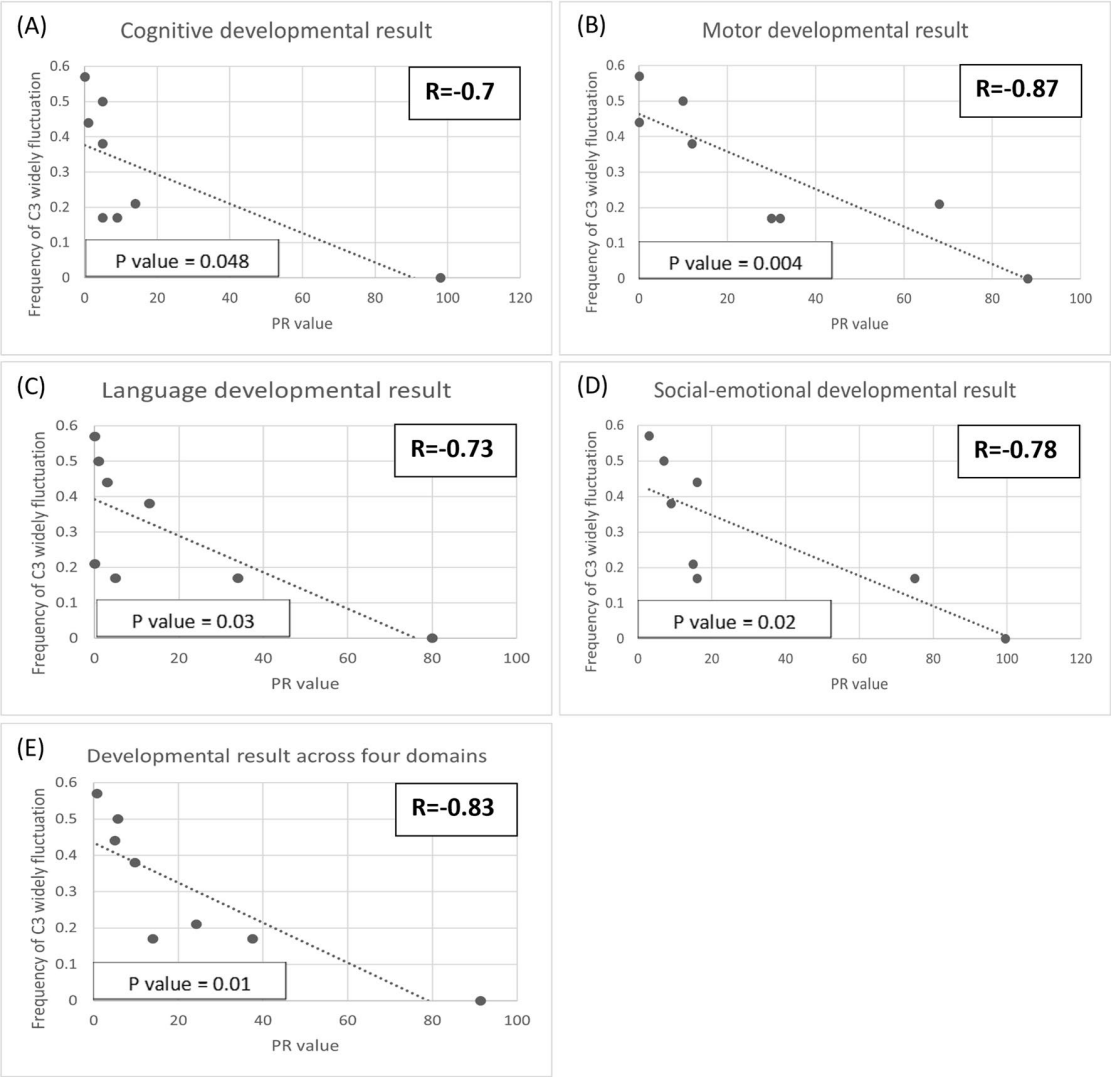
Scatter plot of the PR values from the Bayley III screening test at different frequencies of widely fluctuating C3 (> 1 SD). Each dot represents an individual’s mean frequency of widely fluctuating C3 per blood test and their corresponding PR value. **A** Cognitive development result, **B** Motor development result, **C** Language development result, **D** Social-emotional development result, **E** average development result across all four domains. Strong correlations were found between widely fluctuating C3 levels and all four domains of development, each of which demonstrated statistical significance with *p*-values less than 0.05

**Fig. 4:**
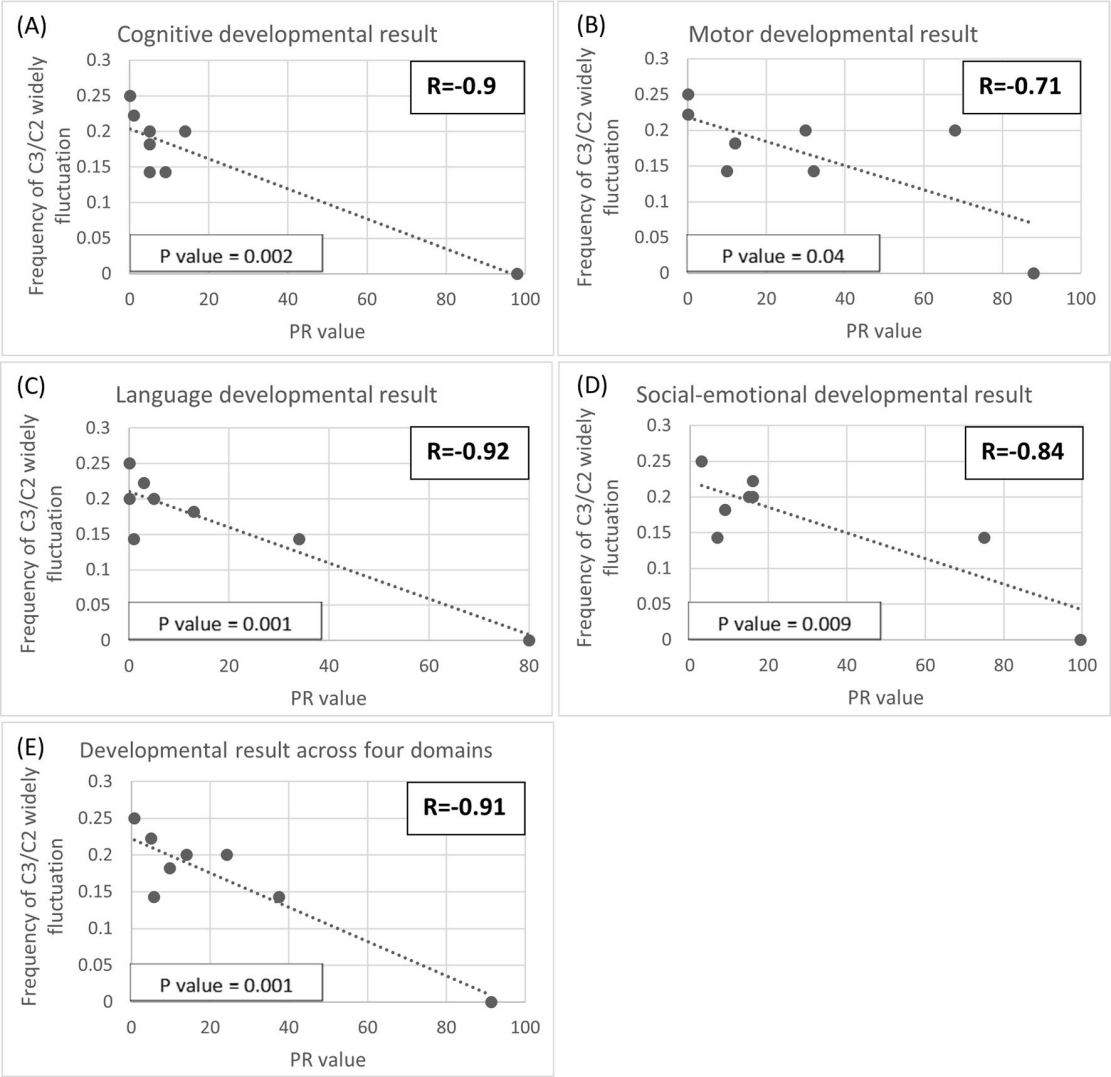
Scatter plot of the PR values from the Bayley III screening test at different frequencies of widely fluctuating C3/C2 (> 1 SD). Each dot represents an individual’s mean frequency of widely fluctuating C3/C2 per blood test and their corresponding PR value. **A** Cognitive development result, **B** Motor development result, **C** Language development result, **D** Social-emotional development result, **E** average development result across all four domains. Strong correlations were found between widely fluctuating C3/C2 levels and all four domains of development, each of which demonstrated statistical significance with *p*-values less than 0.05

To understand the relationship between neurocognitive development and toxic organic acids, we examined the correlation between the frequency of widely fluctuating toxic organic acids and the developmental status of MMA patients. We observed strong negative correlations between the frequency of widely fluctuating MA, C3, and C3/C2 and the cognitive, motor, language, and social-emotional PR values, as well as the average PR value across these four domains (Figs. 2, 3, 4). The Pearson correlation coefficients (r), indicated in each figure, were all above the threshold of 0.7. Moreover, all associated *p*-values, also shown in each figure, were below 0.05, confirming statistical significance. Additionally, strong negative correlations were found between the frequency of widely fluctuating MA, C3, and C3/C2 and the composite scores on the Bayley III screening test. Each correlation demonstrated statistical significance with *p*-values less than 0.05. (Figs. S1–S3).

**Fig. 5:**
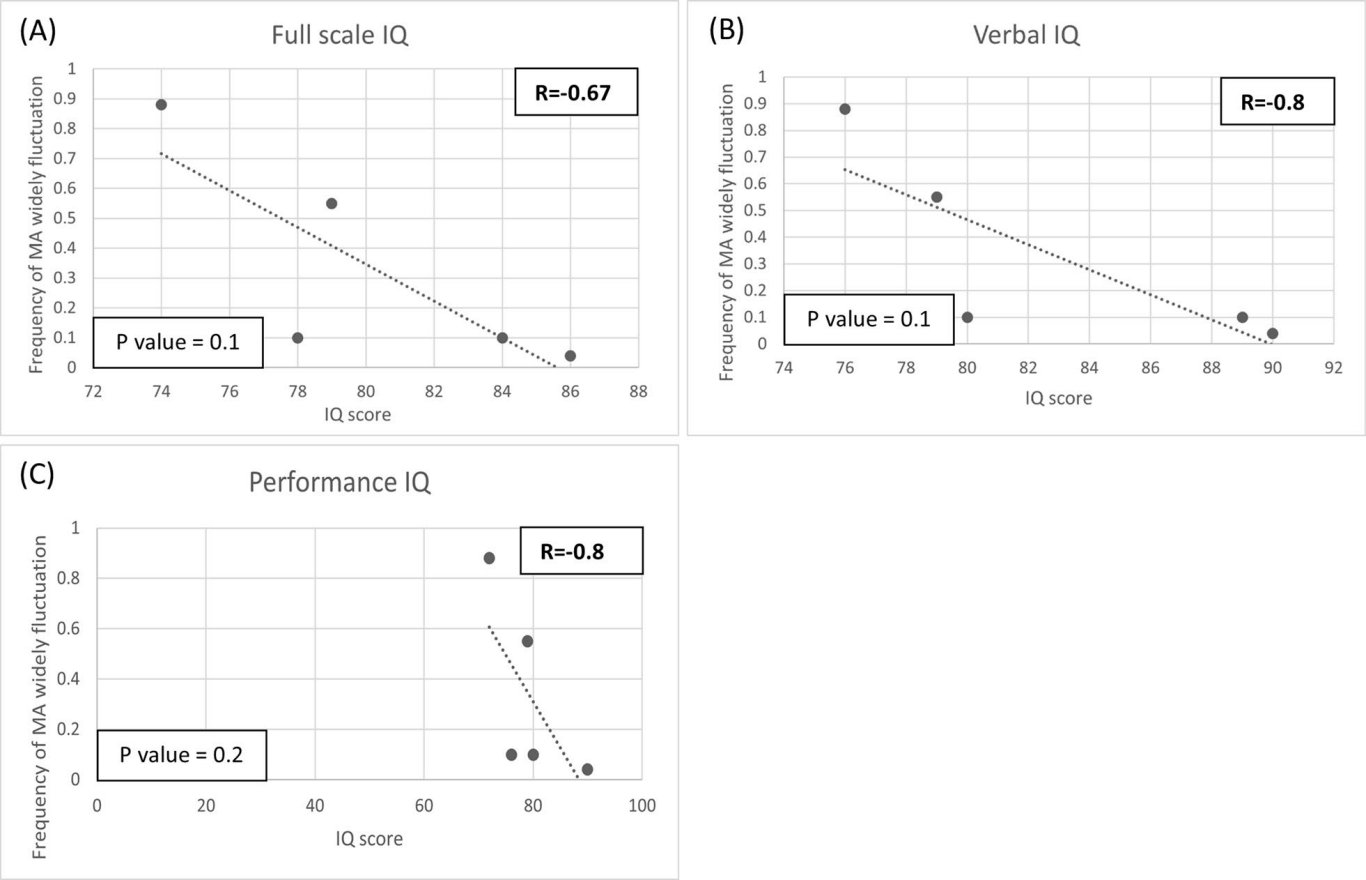
Scatter plot of IQ scores from the Wechsler Intelligence Scale test at different frequencies of MA fluctuation (> 1SD). Each dot represents an individual’s mean frequency of widely fluctuating MA per blood test and their corresponding IQ score. **A** Full scale IQ, **B** Verbal IQ, **C** Performance IQ. Strong correlations exist between widely fluctuating MA and both full-scale IQ and verbal IQ. However, the results did not reach statistical significance, as all *p*-values were above 0.05

**Fig. 6:**
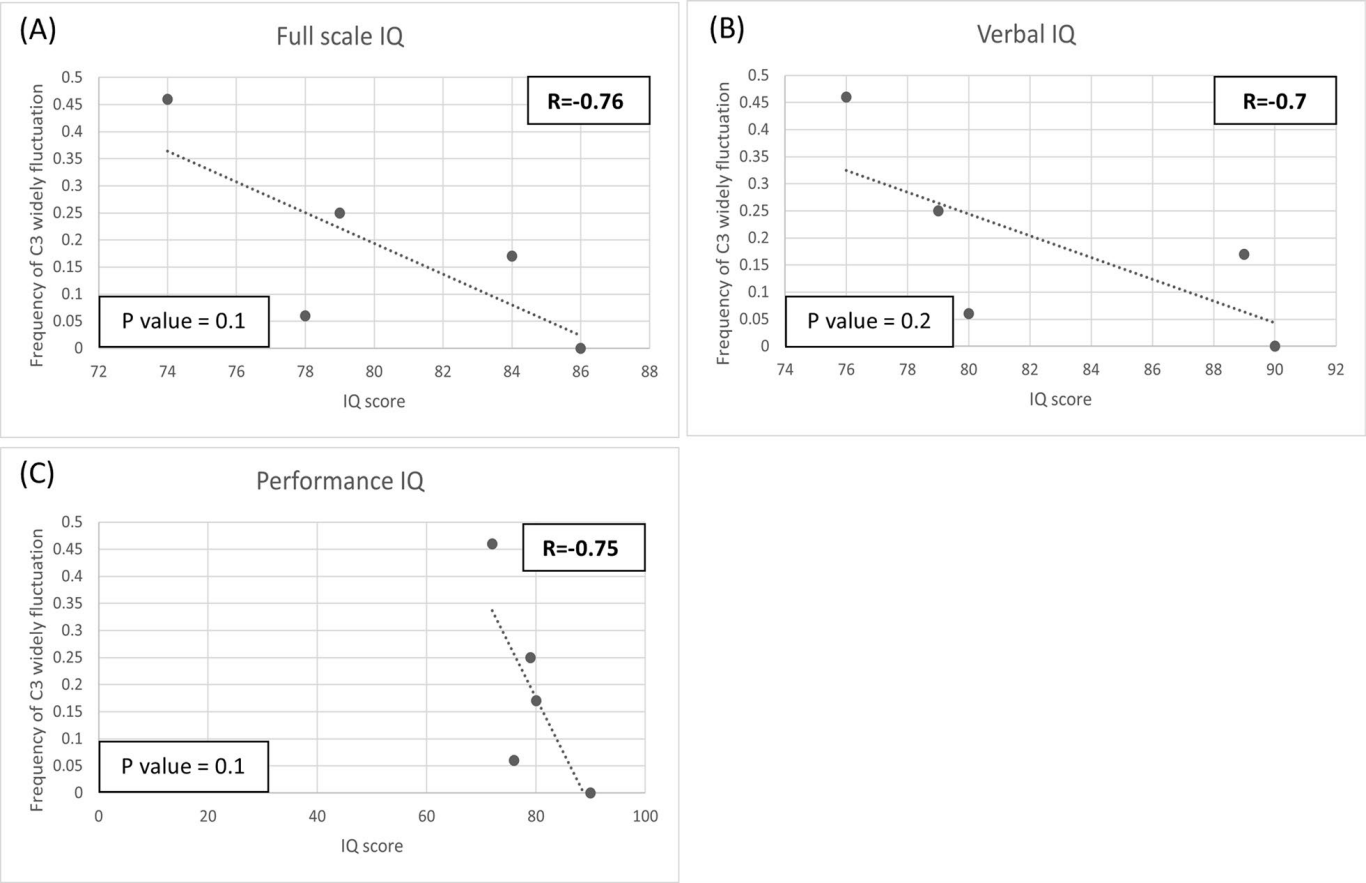
Scatter plot of IQ scores from the Wechsler Intelligence Scale test at different frequencies of C3 fluctuation (> 1SD). Each dot represents an individual’s mean frequency of widely fluctuating C3 per blood test and their corresponding IQ score. **A** Full scale IQ, **B** Verbal IQ, **C** Performance IQ. Strong correlations exist between widely fluctuating C3 and full-scale IQ, verbal IQ, and performance IQ. However, the results did not reach statistical significance, as all *p*-values were above 0.05

**Fig. 7:**
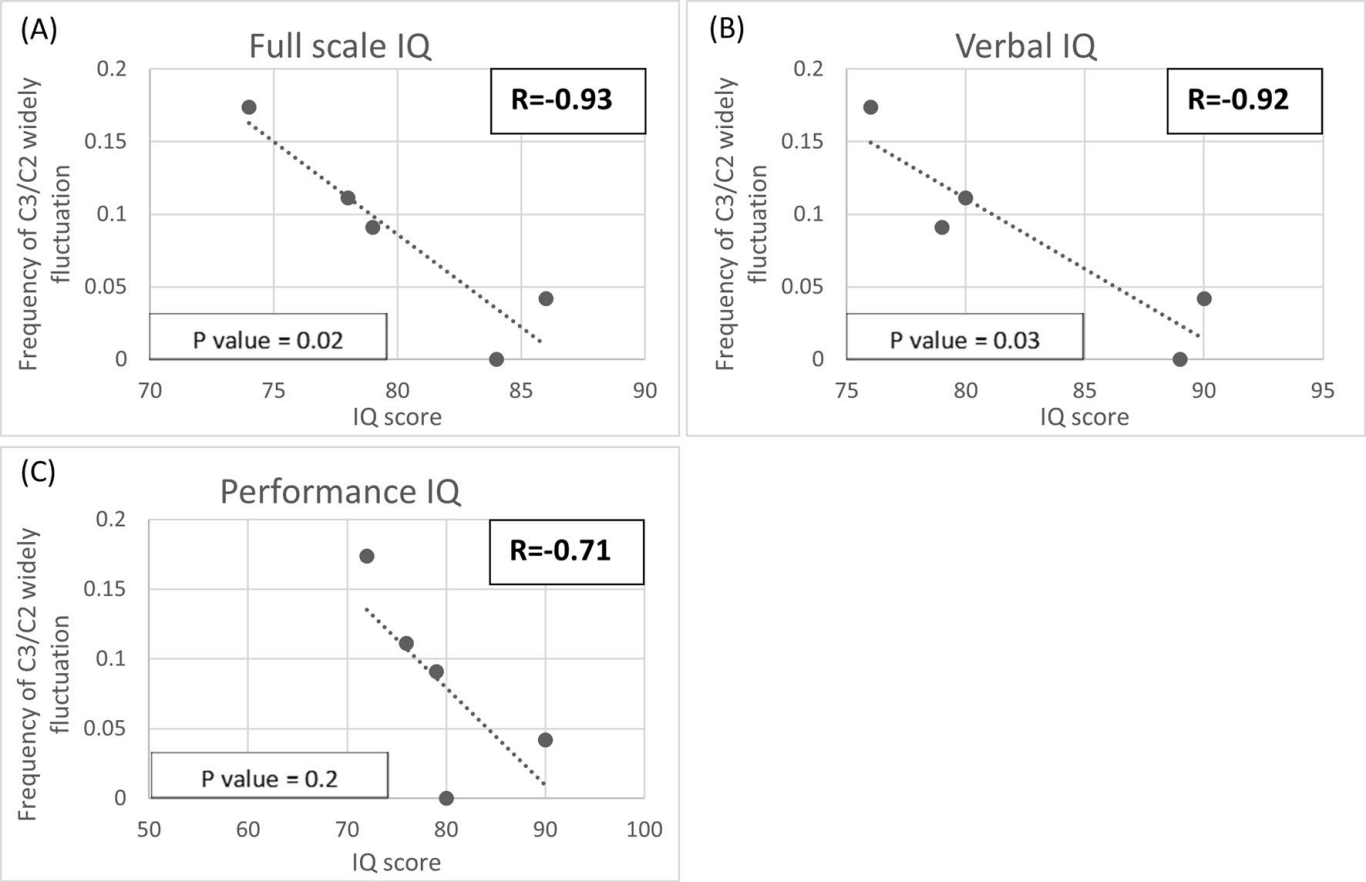
Scatter plot of IQ scores from the Wechsler Intelligence Scale test at different frequencies of C3/C2 fluctuation (> 1SD). Each dot represents an individual’s mean frequency of widely fluctuating C3/C2 per blood test and their corresponding IQ score. **A** Full scale IQ, **B** Verbal IQ, **C** Performance IQ. Strong correlations exist between widely fluctuating C3/C2 and full-scale IQ, verbal IQ, and performance IQ. Both full-scale IQ and verbal IQ showed statistical significance in these correlations

We observed a similar relationship between the frequency of widespread fluctuations in toxic metabolites and IQ scores from the Wechsler Intelligence Scale test results. Excluding Case 9, who had multiple comorbidities related to liver transplantation, we found strong negative correlations between the frequency of widespread fluctuations in MA and IQ scores, with r values of − 0.8 for both full-scale and verbal IQ. However, the correlation for performance IQ was not as strong (r = − 0.67) Despite these correlations, none of the results reached statistical significance. (Fig. 5). Likewise, strong negative correlations were observed between the frequency of widespread fluctuations in C3 and C3/C2 and the IQ scores for full-scale, verbal, and performance, all with r values above the threshold of 0.7 (Figs. 6, 7). Despite these correlations, most results did not reach statistical significance when analyzed for *p*-values, except for the correlations between widely fluctuating C3/C2 and both full-scale IQ and verbal IQ, which did show statistical significance. In contrast, there was no strong correlation between widely fluctuating MCA and the developmental status or IQ scores of MMA patients (Figs.S4–S6).

From another perspective, we investigated the correlations between the amplitude of toxic metabolite fluctuations and the developmental status and IQ scores of patients. Only a few toxic metabolites showed correlations between their fluctuation amplitudes and both developmental status and IQ scores. Specifically, the correlations between MA and motor, social-emotional, and average PR values of developmental status all reached statistical significance. While strong correlations were also observed between MA and full-scale/verbal IQ, as well as between C3 and full-scale IQ, these did not reach statistical significance. (Table S6). These findings suggest that the frequency of toxic metabolite fluctuations has a stronger correlation with the neurodevelopmental outcomes of MMA patients compared to the amplitude of these fluctuations.

## Discussion

Collective evidence from various studies consistently indicates that MMA is linked with neurological symptoms and damage. The diverse clinical range of neurological symptoms associated with MMA encompass developmental delays, intellectual disabilities, seizures, movement disorders, psychiatric issues, and potentially life-threatening encephalopathy during metabolic crises [2, 7, 9, 13, 18–20]. The severity and onset of these neurological symptoms often align with the degree of metabolic derangement, highlighting the critical importance of early diagnosis and intervention [9]. Numerous prior studies consistently identify developmental delays as one of the most prevalent neurological symptoms in MMA [21–23]. Recent research by Manoli et al.[2] demonstrated a correlation between higher levels of MA and more pronounced developmental delays and cognitive impairments in pediatric patients with MMA. Similarly, Carrillo-Carrasco et al. [24] found a positive correlation between elevated MA levels and an increased risk of seizures in affected individuals. Similarly, a study by Baulny et al. [25] emphasized the potential reversibility of neurological deficits when MA levels are effectively controlled, underlining the imperative nature of timely intervention and metabolic management. These findings underscore the pivotal role of MA as a neurotoxic metabolite and suggest that its accumulation leads to neurological dysfunction [2, 13]. Therefore, monitoring and managing MA levels through diet modifications, vitamin B12 supplementation, and other therapeutic interventions become important to prevent metabolic crises and alleviate the severity of neurological symptoms.

MMA manifests as a metabolic disorder with discernible features in brain magnetic resonance imaging (MRI), offering valuable insights into its neurological implications. These distinctive observations include characteristic changes in the basal ganglia, evident as hyperintensities on T2-weighted images [2, 26–28]. Consistent with prior research, our study aligns with the documented occurrence of demyelination in the CNS among MMA patients, a phenomenon previously explored in studies such as the work by Manoli et al.[2]. In our investigation, we observed that 11 out of 20 MMA patients exhibited demyelination in the CNS.

Our study underscores the presence of developmental delays across multiple domains, encompassing cognitive, motor, language, and social-emotional scales, in individuals with MMA. Specifically, the mean PR values derived from Bayley-III screening tests for all MMA patients reveal significant deficits in cognitive function (PR = 17), motor function (PR = 30), language function (PR = 17), and social-emotional scales (PR = 30). Notably, all these PR values fall below the 50th percentile, emphasizing the severity of developmental delay in individuals with MMA. These study results align with prior research findings, consistent with several studies associating MMA with developmental delays [2, 21–23]. In addition to developmental delays, MMA has shown variable impacts on IQ among affected individuals [10, 13, 19, 29]. Similarly, in our study, a patient was diagnosed with profound intellectual disability at the age of 11. The average PR value of FSIQ of our MMA patients was 11. These findings reinforce the intricate connection between MMA and neurocognitive deficits, highlighting the importance of early intervention and comprehensive care for affected individuals.

In our study, brain imaging assessments in B12-R patients showed more normal results than B12-NR patients, and their neurocognitive function outperformed that of B12-NR patients. None of the B12-R patients exhibited brain demyelination, in contrast to 11 out of 14 B12-NR patients who displayed this condition. When assessing developmental delay using the Bayley-III screening test, B12-R patients demonstrated higher PR values across cognitive, motor, language, and social-emotional scales compared to B12-NR patients, indicative of more favorable developmental outcomes for B12-R patients. In the Wechsler Intelligence Scale test, only one B12-NR patient exhibited profound intellectual disability, with all others scoring within the normal range. However, the average FSIQ for B12-R patients was 85 (PR value: 14.5), exceeding the average score for B12-NR patients, which was 63 (PR value: 6.8). Our study’s results align with established research, notably the study conducted by Colin J. O’Shea et al. in 2012 [13]. Their research indicated that cognitive deficits were more pronounced among patients with early-onset MMUT, while patients with other subtypes exhibited relatively normal cognitive outcomes. Early-onset MMUT patients demonstrated a mean FSIQ of 71.1 ± 14.75, whereas patients with CblA or CblB variants displayed mean FSIQ of 100.7 ± 10.95 and 96.6 ± 10.92, respectively [13].

In contrast to B12-NR patients, our study unveiled significantly reduced levels of MA, MCA and C3 in individuals with B12-R MMA. Prior research has clarified that patients responding to cobalamin typically exhibit lower levels of MA and MCA [2, 23, 24]. Conversely, patients unresponsive to cobalamin often maintain persistently elevated MA and MCA levels despite treatment attempts [1, 25]. It is important to account for the impact of L-carnitine supplementation on C3 levels. This supplementation likely explains the majority of the observed differences. The C3/C2 ratio, which adjusts for the influence of L-carnitine, consequently shows no significant difference between the groups. While elevated C3 levels indicate metabolic disturbance, the C3/C2 ratio corrects for L-carnitine supplementation, allowing for a more precise comparison of metabolic status between different treatment groups.

These findings may be connected to the observed normal brain MRS patterns and superior neurocognitive outcomes in our B12-R patients compared to B12-NR patients. For instance, Case 5, the sole B12-R patient classified as developmentally delayed in cognitive function, displayed elevated levels of MA, MCA, and C3/C2 ratios compared to Case 4, who demonstrated normal neurocognitive development, as well as the entire B12-R group.

Moreover, our study has concentrated on examining the consequences of fluctuations in toxic organic acids, potentially exerting a significant influence on the neurodevelopmental outcomes of MMA patients—an aspect not previously explored in the current literature. For all MMA patients, we identified strong negative correlations, as well as statistical significance, between the frequency of widespread fluctuations in MA, C3, and C3/C2 and developmental status across cognitive, motor, language, and social-emotional scales. Widespread fluctuations in MA, C3 and C3/C2 are also negatively correlated with IQ scores. Although most results did not reach statistical significance when analyzed for *p*-values, the correlations between widely fluctuating C3/C2 and both full-scale IQ and verbal IQ did show significance. This may be due to the limitation of a small sample size.

Differences between B12-NR and B12-R MMA patients manifest not only in absolute values but also in the frequency of widely fluctuating toxic organic acids. These findings strongly imply a connection to the disparities in brain imaging results and neurocognitive functioning observed between these two groups. As previously mentioned, the more frequent the widely fluctuating MA, C3 and C3/C2 experienced by an MMA patient, the higher the likelihood of developmental delays and lower IQs. These distinctions are attributed to diverse treatment approaches. B12-NR patients primarily rely on dietary control with protein restriction. However, striking the right balance between effectively managing toxic organic acids and meeting the patient’s nutritional needs can pose a challenge [2, 30, 31]. Moreover, adherence to the dietary regimen may be problematic for some patients, potentially contributing to the instability of toxic organic acid levels. In contrast, hydroxycobalamin has been proven effective in treating B12-R MMA. All B12-R MMA patients in our study exhibited well-controlled urinary organic acid levels and relatively low-amplitude fluctuations in toxic metabolites. They also demonstrated a normal brain myelination status and better neurocognitive functioning throughout the follow-up period. The concept that widely fluctuating C3 and C3/C2 correlate with developmental delays and lower IQs in MMA patients may be further applied to propionic acidemia patients.

On the other hand, Case 9, a B12-NR individual with MMA, demonstrated the lowest FSIQ among B12-NR patients in our study and was diagnosed with profound intellectual disability. Notably, this patient presented elevated serum ammonia levels, surpassing the average levels observed in B12-NR patients. This rise in ammonia levels could be linked to the patient’s liver transplant, which subsequently unveiled the presence of Dubin-Johnson syndrome. This discovery aligns with findings from various studies [10, 24]. These studies have emphasized the detrimental impact of elevated ammonia levels on cognitive function in individuals with MMA.

While multiple comparison adjustments are generally applied to control false positives, our study intentionally refrained from such adjustments. With methylmalonic acidemia being a rare disease and our sample size inherently limited, multiple comparison corrections would compromise statistical power. This study serves an exploratory purpose, aiming to reveal potential associations that warrant further investigation. Thus, we believe this unadjusted approach is justified to maximize the discovery of relevant trends, which could be further validated in larger studies in the future.

### Limitation

The limitations of our study include a small dataset, likely due to the rarity of the disease. Additionally, a longer follow-up period is necessary to gather sufficient data and track the neurological outcomes of the participants.

## Conclusions

In our study on MMA, distinctive outcomes were observed in two MMA subtypes. Patients with B12-R MMA, undergoing treatment with hydroxycobalamin, displayed lower organic acid levels and less frequent fluctuations, leading to better neurocognitive outcomes and improved brain imaging results. In contrast, patients with B12-NR MMA encountered greater challenges, with higher organic acid levels, frequent fluctuations, brain demyelination, and poorer neurocognitive development. Strong negative correlations were identified between the frequency of widely fluctuating MA, C3, C3/C2 and both the developmental status and IQ scores in MMA patients. Overall, our findings underscore the significance of addressing organic acid fluctuations and tailoring treatments for MMA subtypes to improve neurodevelopmental outcomes.

## Supplementary Information

Supplementary material 1. Figure S1. Scatter plot of the composite scores from the Bayley III screening test at different frequencies of widely fluctuating MA (>1 SD). Each dot represents an individual's mean frequency of widely fluctuating MA per blood test and their corresponding composite score. A) Cognitive development result, B) Motor development result, C) Language development result, D) Social-emotional development result, E) average development result across all four domains. Significant correlations were identified between the widely fluctuating MA levels and all four domains of development, with each showing statistical significance at p-values below 0.05.

Supplementary material 2. Figure S2. Scatter plot of the composite scores from the Bayley III screening test at different frequencies of widely fluctuating C3 (>1 SD). Each dot represents an individual's mean frequency of widely fluctuating C3 per blood test and their corresponding composite score. A) Cognitive development result, B) Motor development result, C) Language development result, D) Social-emotional development result, E) average development result across all four domains. Strong correlations were found between widely fluctuating C3 levels and all four domains of development, each of which demonstrated statistical significance with p-values less than 0.05.

Supplementary material 3. Figure S3. Scatter plot of the composite scores from the Bayley III screening test at different frequencies of widely fluctuating C3/C2 (>1 SD). Each dot represents an individual's mean frequency of widely fluctuating C3/C2 per blood test and their corresponding composite score. A) Cognitive development result, B) Motor development result, C) Language development result, D) Social-emotional development result, E) average development result across all four domains. Strong correlations were found between widely fluctuating C3/C2 levels and all four domains of development, each of which demonstrated statistical significance with p-values less than 0.05.

Supplementary material 4. Figure S4. Scatter plot of the PR values from the Bayley III screening test at different frequencies of widely fluctuating MCA (>1 SD). Each dot represents an individual's mean frequency of widely fluctuating MCA per blood test and their corresponding PR value. A) Cognitive development result, B) Motor development result, C) Language development result, D) Social-emotional development result, E) average development result across all four domains. No strong correlations or statistical significance were found.

Supplementary material 5. Figure S5. Scatter plot of the composite scores from the Bayley III screening test at different frequencies of widely fluctuating MCA (>1 SD). Each dot represents an individual's mean frequency of widely fluctuating MCA per blood test and their corresponding composite score. A) Cognitive development result, B) Motor development result, C) Language development result, D) Social-emotional development result, E) average development result across all four domains. No strong correlations or statistical significance were found.

Supplementary material 6. Figure S6. Scatter plot of IQ scores from the Wechsler Intelligence Scale test at different frequencies of MCA fluctuation (> 1SD). Each dot represents an individual's mean frequency of widely fluctuating MCA per blood test and their corresponding IQ score. A) Full scale IQ, B) Verbal IQ, C) Performance IQ. No strong correlation or statistical significance were found.

Supplementary material 7.

## Data Availability

Data sharing is not applicable to this article as no new data were created or analyzed in this study.

## Abbreviations

MMA: Methylmalonic acidemia
MMUT: Methylmalonyl-CoA mutase
cbl: Cobalamin
MA: Methylmalonic acid
MCA: Methylcitric acid
C3: Propionylcarnitine
C2: Acetylcarnitine
MRS: Magnetic resonance spectroscopy
TCA: Tricarboxylic acid
LT: Liver transplantation
SD: Standard deviation
IQ: Intelligence quotient
PR: Percentile rank
CS: Composite score
FSIQ: Full-scale intelligence quotient
MRI: Magnetic resonance imaging
CNS: Central nervous system
B12-NR: Vitamin B12 non-responsive
B12-R: Vitamin B12 responsive
