# Supplementary figures and images for: Correlation between toxic organic acid fluctuations and neurodevelopment in patients with methylmalonic acidemia
Source: Orphanet J Rare Dis. 2025 Apr 15;20:179. doi: 10.1186/s13023-025-03687-3 (PMC11998238; doi:10.1186/s13023-025-03687-3)

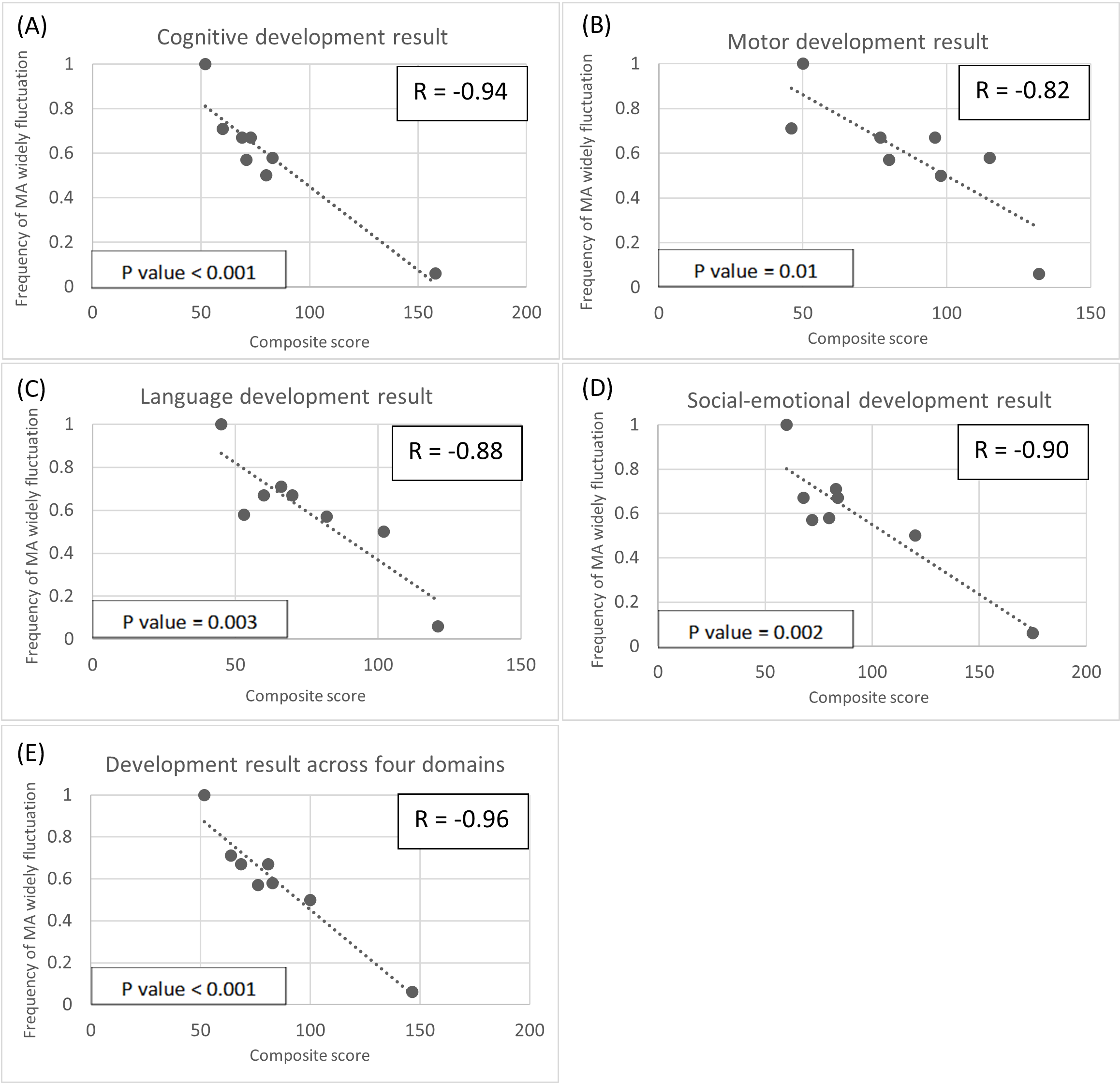

Supplement: Supplementary file 1 — Supplementary material 1. Figure S1. Scatter plot of the composite scores from the Bayley III screening test at different frequencies of widely fluctuating MA (>1 SD). Each dot represents an individual's mean frequency of widely fluctuating MA per blood test and their corresponding composite score. A) Cognitive development result, B) Motor development result, C) Language development result, D) Social-emotional development result, E) average development result across all four domains. Significant correlations were identified between the widely fluctuating MA levels and all four domains of development, with each showing statistical significance at p-values below 0.05. [file 13023_2025_3687_MOESM1_ESM.tif]

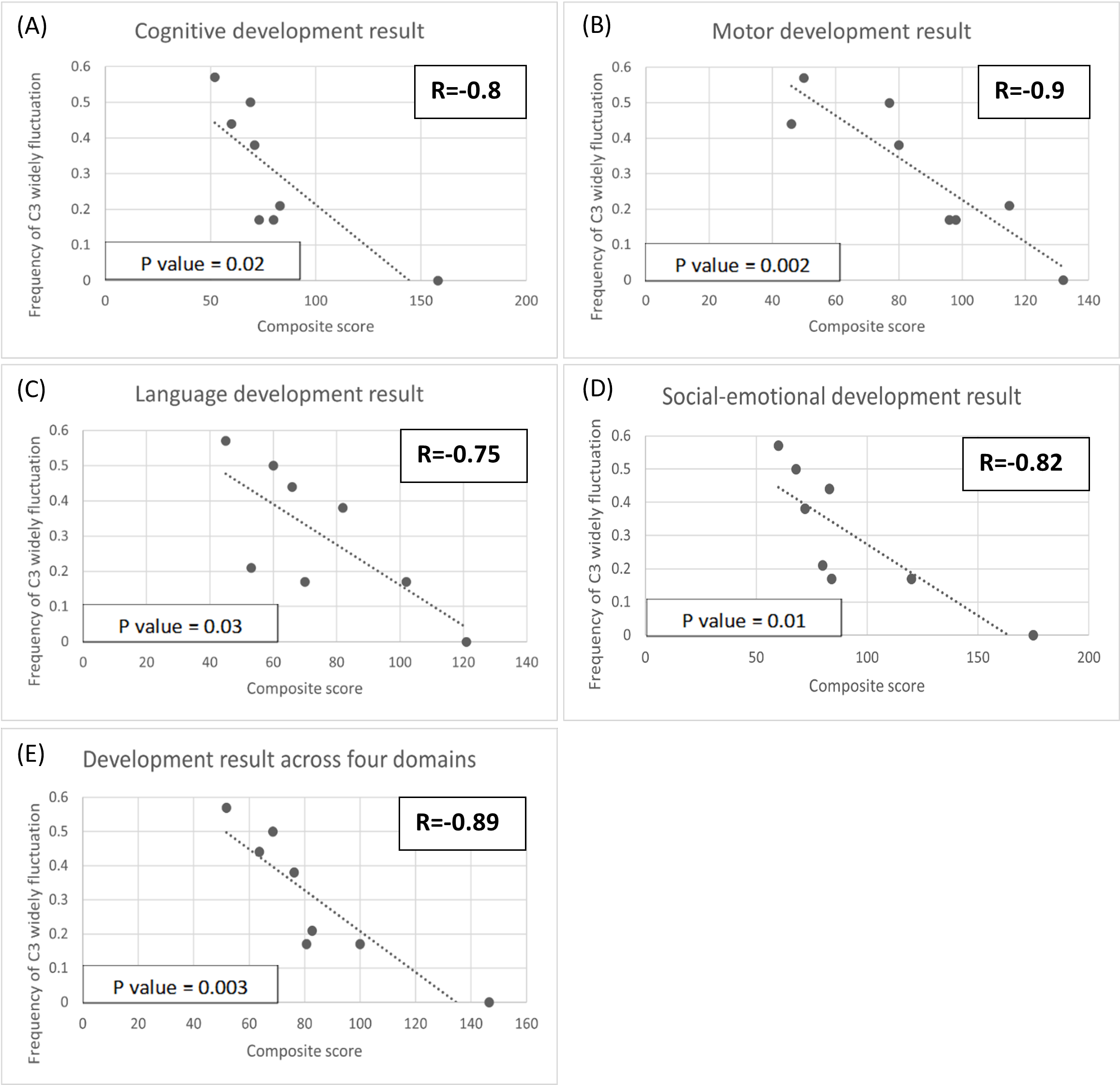

Supplement: Supplementary file 2 — Supplementary material 2. Figure S2. Scatter plot of the composite scores from the Bayley III screening test at different frequencies of widely fluctuating C3 (>1 SD). Each dot represents an individual's mean frequency of widely fluctuating C3 per blood test and their corresponding composite score. A) Cognitive development result, B) Motor development result, C) Language development result, D) Social-emotional development result, E) average development result across all four domains. Strong correlations were found between widely fluctuating C3 levels and all four domains of development, each of which demonstrated statistical significance with p-values less than 0.05. [file 13023_2025_3687_MOESM2_ESM.tif]

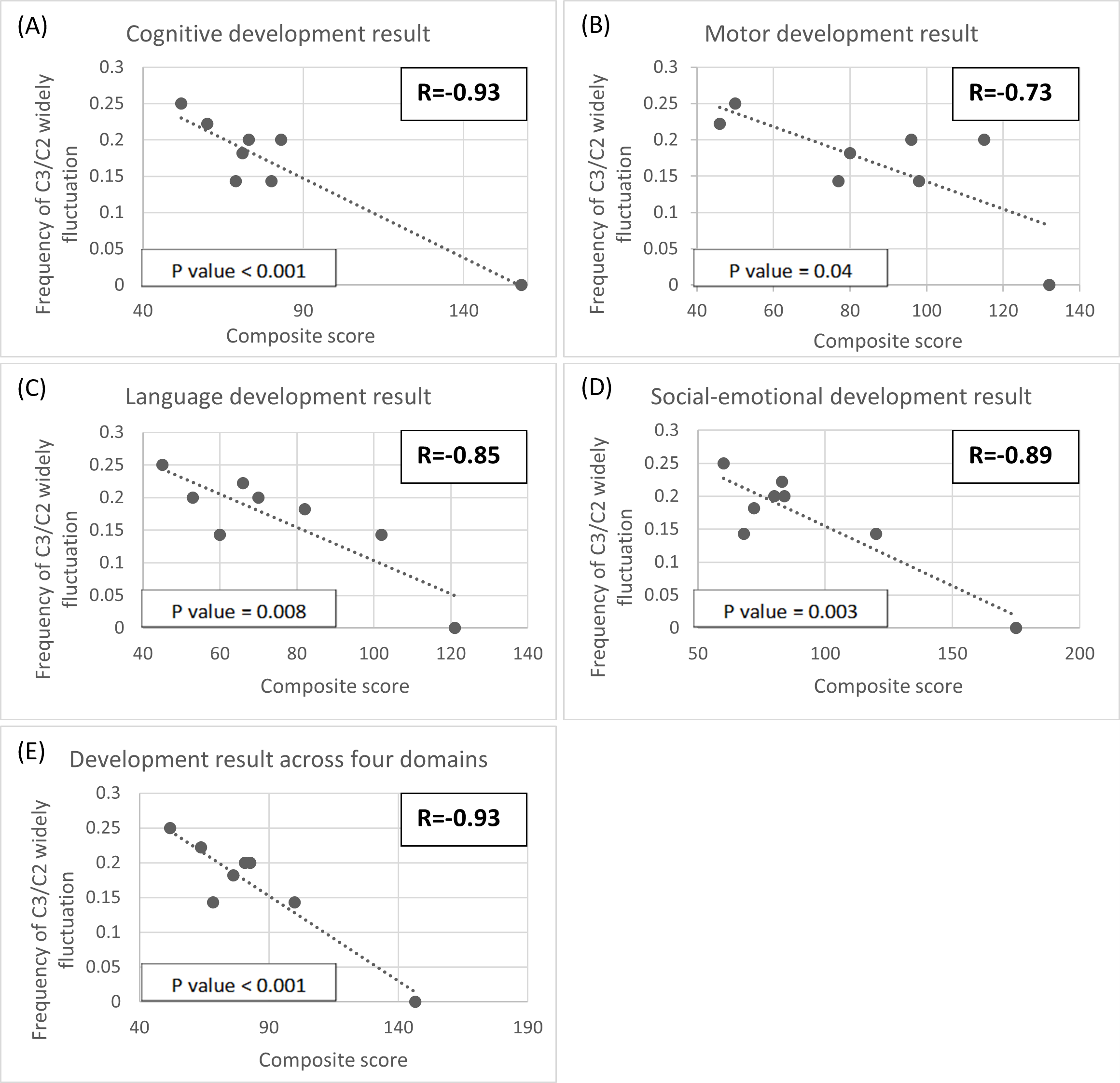

Supplement: Supplementary file 3 — Supplementary material 3. Figure S3. Scatter plot of the composite scores from the Bayley III screening test at different frequencies of widely fluctuating C3/C2 (>1 SD). Each dot represents an individual's mean frequency of widely fluctuating C3/C2 per blood test and their corresponding composite score. A) Cognitive development result, B) Motor development result, C) Language development result, D) Social-emotional development result, E) average development result across all four domains. Strong correlations were found between widely fluctuating C3/C2 levels and all four domains of development, each of which demonstrated statistical significance with p-values less than 0.05. [file 13023_2025_3687_MOESM3_ESM.tif]

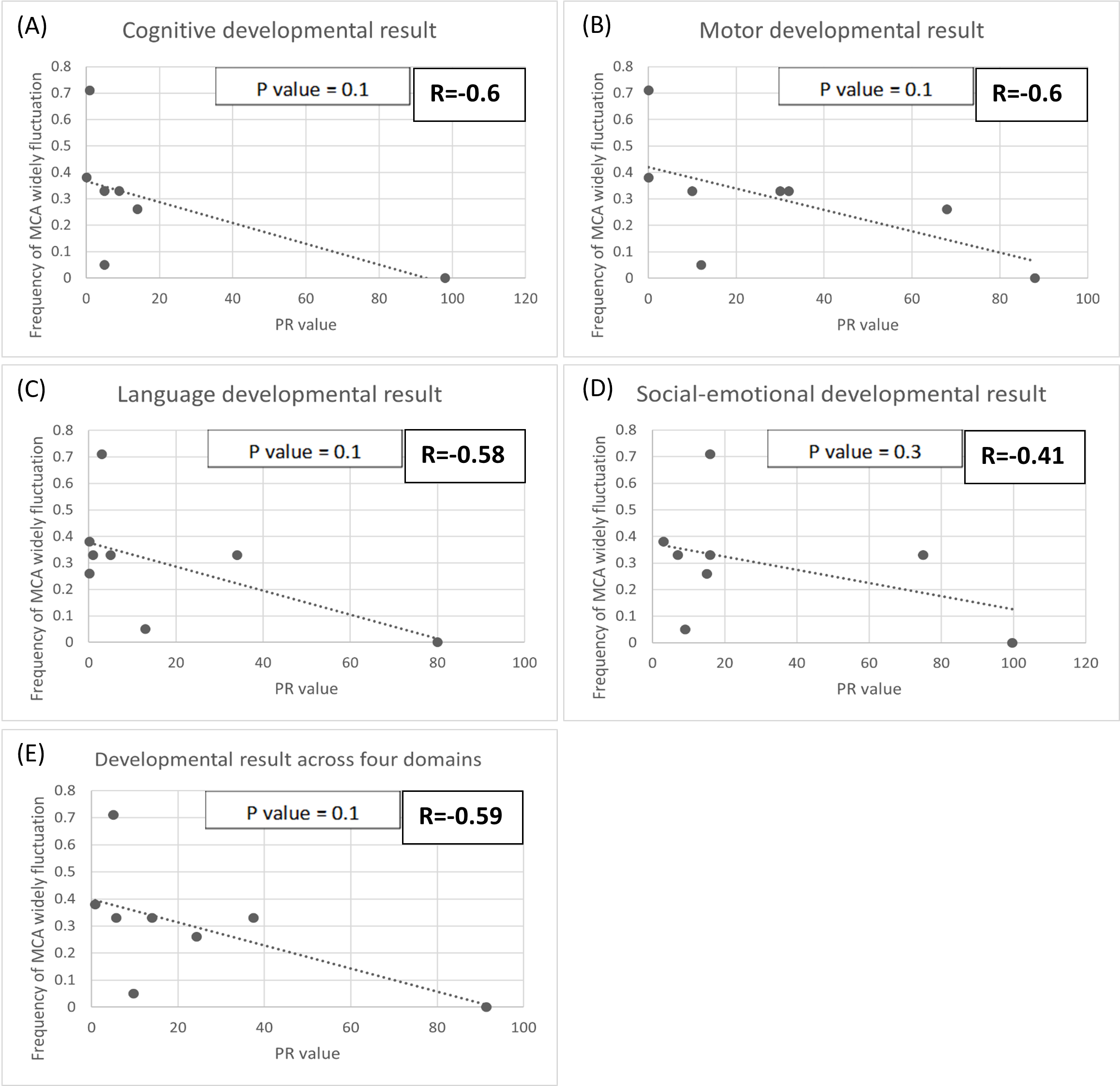

Supplement: Supplementary file 4 — Supplementary material 4. Figure S4. Scatter plot of the PR values from the Bayley III screening test at different frequencies of widely fluctuating MCA (>1 SD). Each dot represents an individual's mean frequency of widely fluctuating MCA per blood test and their corresponding PR value. A) Cognitive development result, B) Motor development result, C) Language development result, D) Social-emotional development result, E) average development result across all four domains. No strong correlations or statistical significance were found. [file 13023_2025_3687_MOESM4_ESM.tif]

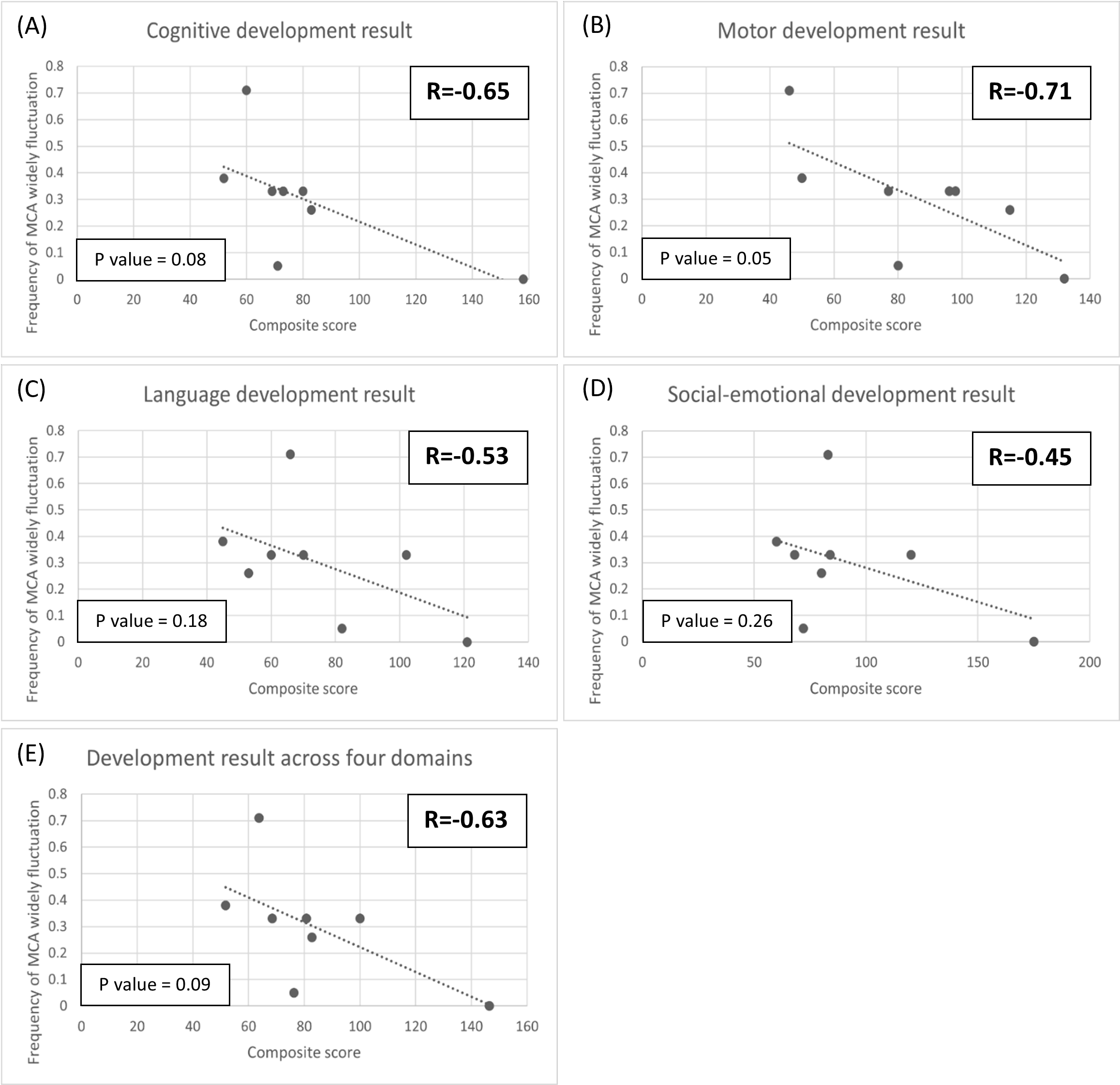

Supplement: Supplementary file 5 — Supplementary material 5. Figure S5. Scatter plot of the composite scores from the Bayley III screening test at different frequencies of widely fluctuating MCA (>1 SD). Each dot represents an individual's mean frequency of widely fluctuating MCA per blood test and their corresponding composite score. A) Cognitive development result, B) Motor development result, C) Language development result, D) Social-emotional development result, E) average development result across all four domains. No strong correlations or statistical significance were found. [file 13023_2025_3687_MOESM5_ESM.tif]

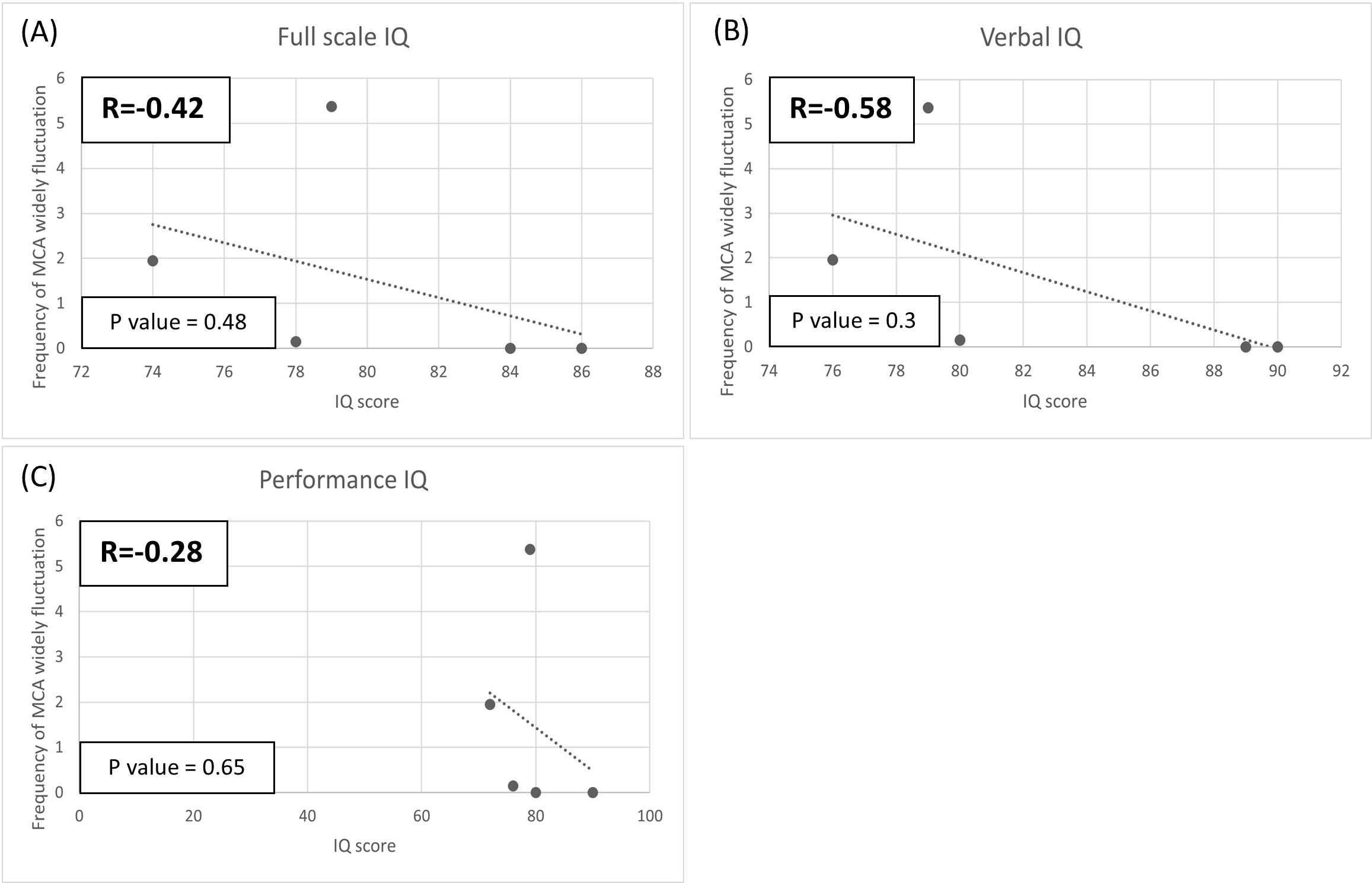

Supplement: Supplementary file 6 — Supplementary material 6. Figure S6. Scatter plot of IQ scores from the Wechsler Intelligence Scale test at different frequencies of MCA fluctuation (> 1SD). Each dot represents an individual's mean frequency of widely fluctuating MCA per blood test and their corresponding IQ score. A) Full scale IQ, B) Verbal IQ, C) Performance IQ. No strong correlation or statistical significance were found. [file 13023_2025_3687_MOESM6_ESM.tif]
