# Supplementary material for: Correlation between toxic organic acid fluctuations and neurodevelopment in patients with methylmalonic acidemia
Source: Orphanet J Rare Dis. 2025 Apr 15;20:179. doi: 10.1186/s13023-025-03687-3 (PMC11998238; doi:10.1186/s13023-025-03687-3)
Supplement: Supplementary file 7 — Supplementary material 7. [file 13023_2025_3687_MOESM7_ESM.docx]

Supplement Tables

Table S1 MRS findings in 15 MMA Study Participants.

|  | MMA subtype^1^ | Brain MRS findings |
| --- | --- | --- |
| Case 1 | B12-R | Normal |
| Case 3 | B12-R | Normal |
| Case 4 | B12-R | Normal |
| Case 6 | B12-R | Normal |
| Case 7 | B12-NR | Elevation of choline complex, white matter dysmyelination |
| Case 8 | B12-NR | Elevation of choline complex, white matter dysmyelination |
| Case 9 | B12-NR | Recent infarct in bilateral cerebral peduncles, encephalomalacia, white matter dysmyelination |
| Case 10 | B12-NR | Elevation of choline complex, white matter dysmyelination |
| Case 11 | B12-NR | Elevation of choline complex, Decreased NAA^2^, white matter dysmyelination |
| Case 12 | B12-NR | Elevation of choline complex, white matter dysmyelination |
| Case 13 | B12-NR | Elevation of choline complex, white matter dysmyelination |
| Case 14 | B12-NR | Elevation of choline complex, white matter dysmyelination |
| Case 16 | B12-NR | External hydrocephalus, elevation of choline complex, white matter dysmyelination |
| Case 17 | B12-NR | Elevation of choline complex, white matter dysmyelination |
| Case 18 | B12-NR | Elevation of choline complex, white matter dysmyelination |
| Case 19 | B12-NR | Elevation of choline complex, white matter dysmyelination |

^1^ B12-R, vitamin B12 responsive; B12-NR, vitamin B12 non-responsive; ^2^ NAA, N-acetylaspartate

. Table S2 Comparing the metabolites between B12-NR patients who have undergone LT versus those who have not.

| Patient | B12-NR  With LT | B12-NR  Without LT | *P* value |
| --- | --- | --- | --- |
| ammonia(ug/dL) | 67.3 ± 45.5 | 75.2 ± 15.3 | 0.7 |
| lactate(mg/dL) | 26.6 ± 7.8 | 37.8 ± 19.3 | 0.22 |
| Urine MA^a^ (μmol/mmol CRE) | 225.8 ± 21 | 266.5 ± 254.6 | 0.71 |
| Urine MCA^b^ (μmol/L) | 12.2 ± 4.9 | 20.6 ± 37.3 | 0.6 |
| Glycine (μmol/L) | 336.3 ± 47.1 | 357.1 ± 44.4 | 0.6 |
| C3^c^ (μM) | 33.5 ± 8.4 | 20 ± 16.4 | 0.11 |
| C3/C2^d^ | 0.86 ± 0.15 | 0.66 ± 0.34 | 0.22 |

^a^ MA, methylmalonic acid; ^b^ MCA, methylcitric acid; ^c^ C3, propionylcarnitine; ^d^ C2, acetylcarnitine

Table S3. Biochemical Markers in Twenty MMA Study Participants

| Case No. | MA^1^  (μmol/mmol CRE) | Frequency of MA wide fluctuation | Average MA^1^ fluctuation range | MCA^2^  (μmol/L) | Frequency of MCA wide fluctuation | Average MCA fluctuation range | | C3^3^  (μM) | Frequency of C3 wide fluctuation | Average C3 fluctuation range | C3/C2^4^ | Ammonia  (ug/dL) | Lactate  (mg/dL) | Glycine  (μmol/L) |
| --- | --- | --- | --- | --- | --- | --- | --- | --- | --- | --- | --- | --- | --- | --- |
| Case 1 | 91.3 | 0.1 | 24.7 | 2.22 | 0 | 2.26 | 15.91 | | 0.17 | 8.09 | 1 | 57.2 | 33.2 | 539.3 |
| Case 2^#^ | 88.4 | - | - | 5.77 | - | - | 9.42 | | - | - | 0.95 | 87 | 61.1 | 252.4 |
| Case 3 | 24.3 | 0.04 | 16.6 | 0.24 | 0 | 0.21 | 3.84 | | 0 | 1.09 | 0.25 | 27.7 | 37.8 | 169 |
| Case 4 | 16.6 | 0.06 | 13.8 | 0.45 | 0 | 0.77 | 5.53 | | 0 | 1.74 | 0.23 | 62.3 | 33.3 | 158.1 |
| Case 5 | 76 | 0.5 | 59.6 | 4.48 | 0.33 | 8.24 | 4.61 | | 0.17 | 2.9 | 1.04 | 36.8 | 39.6 | 147.2 |
| Case 6 | 28.9 | 0 | 28.2 | 3.77 | 0 | 2.8 | 2.94 | | 0 | 2.17 | 0.22 | 49.3 | 37.7 | 166.7 |
| Case 7 | 39.6 | 0.1 | 25.3 | 0.19 | 0 | 0.15 | 9.56 | | 0.06 | 2.81 | 0.44 | 25.7 | 19.5 | 268.4 |
| Case 8 | 218.9 | 0.88 | 179.3 | 4.2 | 0.13 | 1.95 | 41.03 | | 0.46 | 16.73 | 0.69 | 34.1 | 19.4 | 395.8 |
| Case 9 | 107.7 | 0.29 | 42.1 | 8.79 | 0.04 | 4.2 | 39.43 | | 0.52 | 15.18 | 1.19 | 125 | 24.2 | 251.8 |
| Case 10 | 220.7 | 0.55 | 98.9 | 14 | 0.14 | 5.37 | 29.9 | | 0.25 | 8.12 | 0.95 | 75.9 | 18.1 | 361 |
| Case 11 | 261.1 | 0.57 | 140.6 | 11.7 | 0.05 | 4.26 | 47.34 | | 0.38 | 14.95 | 1.07 | 66.1 | 28.2 | 329.4 |
| Case 12 | 214.1 | 0.58 | 119.1 | 12.57 | 0.26 | 6.5 | 35.08 | | 0.21 | 6.94 | 0.88 | 72.2 | 29.5 | 298.3 |
| Case 13 | 241.8 | 0.71 | 220.9 | 8.94 | 0.171 | 6.62 | 31.21 | | 0.44 | 14.86 | 0.84 | 69.9 | 41.3 | 338.5 |
| Case 14 | 527.3 | 1 | 366.2 | 7.93 | 0.38 | 4.37 | 44.3 | | 0.57 | 17 | 0.93 | 54.4 | 27.7 | 323.2 |
| Case 15 | 275.6 | 0.78 | 267.1 | 11.7 | 0.56 | 11.14 | 37.35 | | 0.56 | 15.8 | 1.19 | 84.8 | 35.5 | 228.2 |
| Case 16 | 228.2 | 0.67 | 123.3 | 13.68 | 0.33 | 7.76 | 23.45 | | 0.5 | 11.06 | 0.64 | 79.7 | 27.3 | 257.5 |
| Case 17 | 195.5 | 0.67 | 191.4 | 20 | 0.33 | 11.29 | 26.17 | | 0.17 | 5.61 | 0.97 | 73.5 | 22.5 | 374.2 |
| Case 18 | 33.8 | 0 | 7.45 | 3.97 | 0 | 0.94 | 5.63 | | 0 | 1.28 | 0.26 | 36 | 22.5 | 190.9 |
| Case 19 | 104 | 0.4 | 34.8 | 3.66 | 0 | 2.55 | 11.39 | | 0 | 1.25 | 0.51 | 104.2 | 52.6 | 259.3 |
| Case 20^#^ | 618.7 | - | - | 96.34 | - | - | 11.98 | | - | - | 0.63 | 146 | 68.9 | 240 |

^1^ MA, methylmalonic acid; ^2^ MCA, methylcitric acid; ^3^ C3, propionylcarnitine; ^4^ C2, acetylcarnitine

^#^ Case 2 and Case 20, both aged 0.33 years during data collection, lack variability data for MA, MCA, and C3, as we calculated absolute differences between two blood test results obtained six months apart.

Table S4 Variability in Biomarker Levels Between B12-R and B12-NR Patients Using Unpaired t-test and ANOVA

|  | *P* value  (t-test) | *F value*  *(ANOVA)* | *P value*  *(ANOVA)* |
| --- | --- | --- | --- |
| Frequency of widely fluctuating MA # | 0.01 | 8.37 | 0.01 |
| Average MA fluctuation range (μmol/mmol CRE) | <0.0001 | 5.36 | 0.03 |
| Frequency of widely fluctuating MCA | 0.15 | 1.74 | 0.2 |
| Average MCA fluctuation range (μmol/L) | <0.0001 | 6.23 | 0.02 |
| Frequency widely fluctuating C3 | 0.01 | 6.21 | 0.02 |
| Average C3 fluctuation range (μM) | <0.0001 | 5.87 | 0.03 |
| Frequency widely fluctuating C3/C2 | 0.01 | 4.54 | 0.04 |
| Average C3/C2 fluctuation range | 0.79 | 3.88 | 0.72 |

Table S5 Comparing the variability of metabolites between B12 non-responsive (B12-NR) patients who have undergone liver transplantation (LT) and those who have not.

|  | B12-NR  With LT | B12-NR  Without LT | *p* value |
| --- | --- | --- | --- |
| Frequency of widely fluctuating MA ^#^ | 0.66 ± 0.11 | 0.46 ± 0.43 | 0.35 |
| Average MA fluctuation range (μmol/mmol CRE) | 153.4 ± 44.5 | 140.2 ± 165.2 | 0.87 |
| Frequency of widely fluctuating MCA | 0.19 ± 0.27 | 0.2 ± 0.11 | 0.92 |
| Average MCA fluctuation range (μmol/L) | 6.25 ± 2.92 | 3.83 ± 4.4 | 0.32 |
| Frequency widely fluctuating C3 | 0.34 ± 0.13 | 0.24 ± 0.3 | 0.49 |
| Average C3 fluctuation range (μM) | 11.2 ± 4.4 | 7.6 ± 8 | 0.41 |

MA: methylmalonic acid; MCA: methylcitric acid; C3: propionylcarnitine

# Frequency of wide fluctuation for each biomarker is normalized to total number of blood tests.

Table S6 Pearson Correlation Coefficients (r) and Significance (*p-value*) Between Amplitude of Toxic Metabolite Fluctuations and Patients' Developmental Status and IQ Scores

|  | MA fluctuation amplitude | MCA fluctuation amplitude | C3 fluctuation amplitude | C3/C2 fluctuation amplitude |
| --- | --- | --- | --- | --- |
| Cognitive PR value | -0.6 (0.11)^#^ | -0.67 (0.07) | -0.56 (0.15) | -0.35 (0.4) |
| Motor PR value | -0.76 (0.03) | -0.35 (0.4) | -0.69 (0.06) | -0.35 (0.4) |
| Language PR value | -0.69 (0.06) | -0.59 (0.12) | -0.61 (0.1) | -0.61 (0.1) |
| Social-emotional PR value | -0.74 (0.04) | -0.39 (0.34) | -0.64 (0.09) | -0.26 (0.5) |
| Average PR value | -0.76 (0.03) | -0.53 (0.18) | -0.63 (0.09) | -0.07 (0.87) |
| Full scale IQ | -0.81 (0.09) | -0.22 (0.72) | -0.71 (0.18) | -0.07 (0.91) |
| Verbal IQ | -0.78 (0.12) | -0.34 (0.58) | -0.61 (0.27) | -0.13 (0.83) |
| Performance IQ | -0.67 (0.22) | -0.23 (0.71) | -0.63 (0.25) | -0.28 (0.65) |

# number in parentheses indicates the *p-value*
